# RNA Export through the NPC in Eukaryotes

**DOI:** 10.3390/genes6010124

**Published:** 2015-03-20

**Authors:** Masumi Okamura, Haruko Inose, Seiji Masuda

**Affiliations:** Division of Integrated Life Science, Graduate School of Biostudies, Kyoto University, Kyoto 606-8502, Japan; E-Mail: inose.haruko.24n@st.kyoto-u.ac.jp

**Keywords:** RNA export, mRNA, Nxf1, Crm1, Xpot, Xpo5

## Abstract

In eukaryotic cells, RNAs are transcribed in the nucleus and exported to the cytoplasm through the nuclear pore complex. The RNA molecules that are exported from the nucleus into the cytoplasm include messenger RNAs (mRNAs), ribosomal RNAs (rRNAs), transfer RNAs (tRNAs), small nuclear RNAs (snRNAs), micro RNAs (miRNAs), and viral mRNAs. Each RNA is transported by a specific nuclear export receptor. It is believed that most of the mRNAs are exported by Nxf1 (Mex67 in yeast), whereas rRNAs, snRNAs, and a certain subset of mRNAs are exported in a Crm1/Xpo1-dependent manner. tRNAs and miRNAs are exported by Xpot and Xpo5. However, multiple export receptors are involved in the export of some RNAs, such as 60S ribosomal subunit. In addition to these export receptors, some adapter proteins are required to export RNAs. The RNA export system of eukaryotic cells is also used by several types of RNA virus that depend on the machineries of the host cell in the nucleus for replication of their genome, therefore this review describes the RNA export system of two representative viruses. We also discuss the NPC anchoring-dependent mRNA export factors that directly recruit specific genes to the NPC.

## 1. Introduction

In eukaryotic cells, nascent mRNAs are transcribed as precursor mRNAs (pre-mRNAs) in the nucleus by RNA polymerase II (RNAPII). pre-mRNAs undergo processing steps to become mature mRNAs and form messenger ribonucleoproteins (mRNPs), complexes of mRNA and proteins that are transported to the cytoplasm through the nuclear pore complex (NPC) and translated into proteins.

During transcription, mRNA processing factors are co-transcriptionally associated with the *C*-terminal domain of RNAPII, which promotes mRNA processing such as 5' capping, splicing, and 3' cleavage and polyadenylation [1]. Trans-acting factors bind to mRNA in a processing-dependent manner so that the fully processed mRNA forms mature mRNP. Failure of mRNA processing results in the formation of defective mRNP that is eventually eliminated through mRNA surveillance systems [2,3,4].

The fully processed mRNP includes an export receptor that allows mRNP to pass through the NPC. Two major export receptors are implicated in two distinct mRNA export pathways across the NPC: a Nxf1-Nxt1/Tap-p15 (Mex67-Mtr2 in yeast) heterodimer and Crm1/Xpo1. Nxf1 harbors an RNA binding domain, but free Nxf1 forms a closed conformation that cannot bind RNA efficiently in human cells. Binding of TRanscription-EXport complex 1 (TREX-1) to Nxf1 causes Nxf1 to adopt an open conformation that can efficiently bind mRNA [5]. The other export receptor, Crm1, is not itself an RNA binding protein; instead, it needs an adaptor protein to export mRNP. Several mRNA binding proteins (e.g., HuR, Nmd3) act as adaptors for Crm1 [6]. Crm1 also functions in the export of unspliced or partially spliced viral transcripts with the support of adaptor proteins such as Rev [7].

The NPC is one of the largest protein complexes in eukaryotic cells, penetrating the inner and outer nuclear membrane. A fully assembled NPC has an estimated molecular mass of ~125 MDa in vertebrates [8]. Its three-dimensional structure is highly conserved among eukaryotic cells. NPCs show an eight-fold rotational symmetry and consist of a nuclear ring, a central transport channel, and eight cytoplasmic fibrils. There is a basket structure extended from the nuclear ring [9]. The proteins that compose the NPC are called nucleoporins (Nups). Ions and small molecules can freely diffuse through the pore, but molecules larger than 40–60 kDa pass through the NPC central channel by association with an export receptor such as Nxf1, Crm1, or other karyopherins. Various RNAs and proteins are exported through the NPC in association with a specific export receptor. In general, protein cargoes associate with Crm1 whereas RNAs interact with several different export receptors according to the type of RNA [6].

The RNA molecules that are exported from the nucleus into the cytoplasm include not only mRNAs, but also ribosomal RNAs (rRNAs), transfer RNAs (tRNAs), small nuclear RNAs (snRNAs), micro RNAs (miRNAs), and some types of viral mRNA. rRNA is a component of a ribosomal subunit and functions in translation. tRNAs, also essential molecules for translation, carry specific amino acids to the ribosome. snRNAs and proteins form small nuclear ribonucleic particles (snRNPs), which are the core component of the spliceosome. pre-mRNA splicing at the spliceosome requires at least five types of snRNP containing U1, U2, U4, U5, and U6 snRNA. Additionally, U3 snRNA is required for rRNA processing [10], and U7 snRNA is required to process histone pre-mRNA [11]. miRNAs are small RNAs of approximately 22 nt in length that function in the regulation of gene expression.

In this review we also briefly introduce the mRNA export system of certain viruses whose genome is transferred into the nucleus as viral RNP. Although the export system of viral mRNA from the nucleus differs according to the type of virus, as examples we will describe the systems of Mason–Pfizer monkey virus (MPMV) and human immunodeficiency virus 1 (HIV-1) in this article.

**Figure 1:**
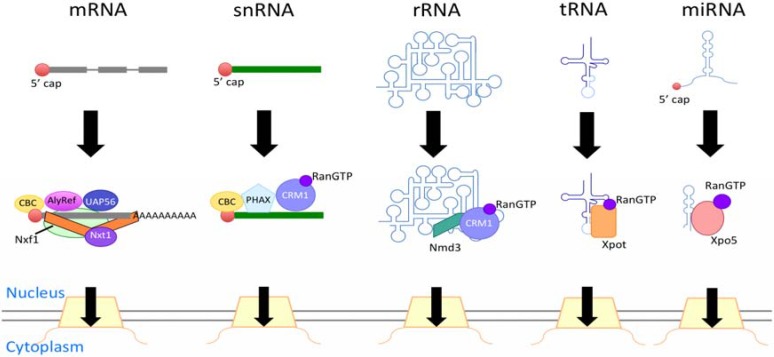
The major routes of RNA export. The transcripts undergo processing and associate with export receptors. This figure shows the representative export receptors for each RNA. CBC: cap-binding complex.

The concept of this review is to introduce the major export pathway of each type of RNA through the NPC (Figure 1). We direct readers to the reviews referenced in this article for more details and information on the minor export pathways of specific RNAs.

## 2. mRNA

### 2.1. Complex Formation for Nxf1-Dependent mRNA Export

The major export receptors of mRNA in mammalian cells are Nxf1 and Crm1. Nxf1 is thought to be the most important mRNA export receptor. Nxf1 forms a heterodimer with Nxt1 that can bind mRNA via TREX-1. TREX-1 is conserved from yeast to humans. Yeast TREX-1 binds mRNA in a transcription-dependent manner, whereas human TREX-1 is recruited to mRNA in a splicing-dependent manner [12,13,14]. Moreover, some studies have shown that several intronless transcripts are exported in a TREX-1- and Nxf1-dependent manner in humans [15,16]. In addition to TREX-1, three SR proteins also function to recruit Nxf1 to spliced mRNA in human cells [17,18]. It is unknown whether the two distinct adaptors differentially regulate the export of specific subsets of mRNAs. Interestingly, Mex67 of *Schizosaccharomyces pombe* is not essential for mRNA export, and overexpression of spMex67p inhibits mRNA export [19]. In the place of spMex67p, Rae1 is required for mRNA export [20].

The function of Nxt1 is still largely unknown. Nxt1 stimulates binding of the Nxf1-RNA complex to nucleoporin p62, and a Nxf1 mutant defective in binding to Nxt1 cannot promote mRNA export in humans [21]. A recent report showed that Nxt1 is required for the expression of testis-specific transcripts in *Drosophila* [22]. Interestingly, it has also been suggested that Nxt1 is involved in Crm1-mediated cargo export. Nxt1 can bind Ran-GTP [23] and promotes the export of tRNA, Crm1-dependent snRNA, and protein cargo *in vitro* [24,25]. These results imply that Nxf1 may support mRNA export via Nxf1- and Crm1-dependent export pathways.

**Figure 2:**
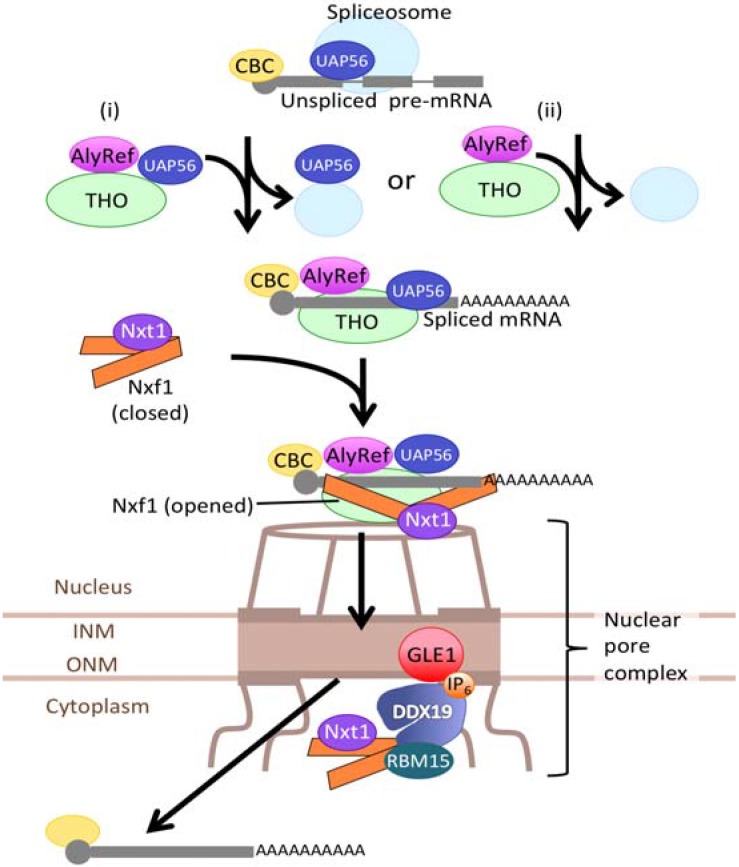
Nxf1-dependent mRNA export. Nascent pre-mRNA is spliced by the spliceosome, and then AlyRef, Uap56, and THO assemble into TREX-1. Uap56 can bind pre-mRNA independently of THO and AlyRef, but it is unclear whether the Uap56 that docks to TREX-1 is distinct from the Uap56 that binds pre-mRNA before binding to TREX-1; *i.e.*, model (i) *versus* model (ii). Spliced mRNA within TREX-1 recruits a NXf1-Nxt1 heterodimer that allows the mRNA to traverse the nuclear pore complex (NPC). INM: inner nuclear membrane, ONM: outer nuclear membrane.

TREX-1 consists of AlyRef (Aly/Thoc4/Ref/Bef), Uap56, Cip29, pDIP3, ZC11A, and the THO subcomplex, which consists of Thoc1 (THO1/Hpr1/p84), Thoc2 (THO2), Thoc3 (THO3/Tex1), Thoc5 (THO5/fSAP79), Thoc6 (THO6/fSAP35), and Thoc7 (THO7/fSAP24). Recruitment of TREX-1 and export of mRNP requires the 5' capping of pre-mRNA because CBP80, the component of the cap binding complex (CBC) that binds on the 5' capping site, interacts with AlyRef and the THO subcomplex [26,27]. Cip29 is also recruited to the mRNA in a cap- and splicing-dependent manner [28]. The DEAD-box RNA helicase Uap56 bridges THO and both AlyRef and Cip29 [28], but can bind pre-mRNA independent of AlyRef and THO. Uap56 is also a component of the TREX-1. It is unclear whether Uap56 binds pre-mRNA before TREX-1 formation and then joins TREX-1, or whether a distinct Uap56 is contained within TREX-1 [27]. TREX-1 is eventually recruited to the 5' end of pre-mRNA. The closed form of Nxf1 has a low affinity for RNA; Thoc5 and AlyRef induce a conformational change from the closed form of Nxf1 to the open form [5]. Binding of Nxf1 to mRNA via TREX-1 allows the mRNA to traverse the NPC (Figure 2). Yra1 of *Saccharomyces cerevisiae* (AlyRef in humans) interacts with the 3' end processing factor Pcf11 [29], and this binding enables coupling between transcription and 3' end processing.

Recently, a novel TREX-1 component was identified and named Chtop [30]. Chtop, one of the chromatin-associated proteins, shows some similarities to AlyRef in its function: it can bind Uap56 competitively with AlyRef and stimulates the ATPase and helicase activity of Uap56. Chtop also promotes the interaction between mRNA and TREX-1. It binds to Nxf1 and then forms a trimeric complex with Thoc5, thus functioning in mRNA export. The Urh49 protein is highly homologous to Uap56, but forms a different complex with Cip29 called AREX and functions in a different mRNA export pathway from that of Uap56 [31]. Whether Urh49-mediated mRNA export depends on Nxf1 is yet to be determined.

Several components of TREX-1 participate in the biogenesis of small RNA. Uap56 functions in the export of Piwi-interacting RNA (piRNA) precursor in *D. melanogaster* [32]. piRNA forms a complex with Piwi protein that functions in the repression of transposable elements in the germline and is considered to maintain germline integrity [33]. Piwi proteins were first identified in *D. melanogaster* [34] and since then Piwi proteins and piRNAs have been identified in *M. musculus*, *R. norvegicus*, and other organisms [33]. In *A. thaliana*, the putative orthologs of Thoc1 and Thoc3 are required for small interfering RNA (siRNA) biosynthesis [35,36]. siRNA functions in the regulation of gene expression by RNA-induced silencing, similar to miRNA [37]. A relationship between siRNA biosynthesis and mRNA export factor is also observed in *S. pombe*; Mlo3, the essential factor for mRNA export in *S. pombe*, is required for siRNA production [38,39]. The molecular mechanism linking mRNA export and the biosynthesis of such small RNAs should be explored in future studies.

### 2.2. Remodeling of mRNP with Nxf1-Nxt1 at the Cytoplasmic Side of NPC

When an mRNP passes through the NPC with nuclear export receptor and enters the cytoplasmic face, a conformational change in the exported mRNP called “remodeling” occurs. This process is essential for mRNA dissociation in the cytoplasm.

The following insights were obtained from studies of *S. cerevisiae*. The main player in the remodeling machinery is the DEAD-box helicase Dbp5 (DDX19 in vertebrates) [40,41,42]. Dbp5 is an ATP-dependent RNA helicase and localizes on the NPC cytoplasmic filament via Nup159 (Nup214 in vertebrates) [43,44]. The RNA helicase activity of Dbp5 is stimulated by the mRNA export factor Gle1 and its cofactor inositol hexakisphosphate (IP_6_) [41,42]. The activity of mRNP remodeling depends on both stimulation by Gle1-IP_6_ and Nup159-triggered ADP release [45,46]. After remodeling, the export receptors and adaptors are re-imported into the nucleus and recycled individually [40,47].

It remains unclear whether these regulatory mechanisms are fully conserved in vertebrates, although the human homologs of Dbp5 and Gle1 can be detected in the cytoplasmic filaments of the NPC and are thought to function in mRNP remodeling. In fact, depletion of DDX19 or hGLE1 results in nuclear accumulation of bulk poly(A) RNA [44,48]. DDX19 and hGLE1 localize at the cytoplasmic side of the nuclear envelope via Nup214 and Nup155, respectively. In addition, RBM15 has been identified as a DDX19 binding factor in humans; RBM15 bridges Nxf1 and DDX19, and probably helps mRNP remodeling [49,50]. Depletion of RBM15 results in the nuclear accumulation of bulk poly(A) RNA [50].

### 2.3. Efficient mRNA Export by the Association of Active Transcription Sites with the NPC

In yeast, the chromatin in the nucleus does not diffuse randomly but is attached to the nuclear membrane. Genome-wide approaches have revealed that some components of the NPC associate with active genes [51,52]. In contrast, heterochromatin (*i.e.*, silenced genes) associates with the inner nuclear membrane. The binding of genes to the NPC requires interaction with the mRNA export machinery and transcribed genes [53]. The relationship between gene localization and the level of expression remains obscure. Artificially targeting a reporter gene to NPCs increases transcriptional output [54], whereas tethering it to the inner nuclear membrane results in transcriptional repression [55]. Other reports show that binding of the transcription machinery to NPC is not essential, and that histone deacetylation is more important than tethering to the nuclear membrane [56,57]. Silenced chromatin was not previously thought to be attached to the NPC; however, a recent finding indicates that the NPC components Mlp1 and Mlp2 interact with silenced chromatin [58], implying that NPC might also anchor heterochromatin. Most studies of the recruitment of active loci to NPC have been performed with yeast, and little is known about this association in higher eukaryotes.

The recruitment of actively transcribed genes to the NPC depends on the association of Spt/Ada/Gcn5 acetyltransferase (SAGA) complex and TREX-2, which functions as an anchor. SAGA is one of the chromatin-modifying transcriptional co-activator complexes and consists of 21 proteins. SAGA thus forces active gene loci to physically contact the NPC via TREX-2. SAGA and TREX-2 are anchored into the nucleoplasmic side of the NPC via the NPC components Mlp1 and Nup1, respectively [56,59]. The deletion of any TREX-2 subunit (Sac3, Thp1, Cdc31, Sus1, or Sem1) results in defective mRNA export in yeast [60,61,62,63,64]. Sus1 is singly recruited to coding regions via the RNAPII *C*-terminal domain.

SAGA was first found in yeast, although homologous proteins have been identified in other eukaryotes. SAGA also functions as an anchor of transcription sites to NPC in *D. melanogaster* [65], in which an ortholog of TREX-2 promotes association of TREX-1 with mRNA [66]. A homolog of TREX-2 has also been identified in *A. thaliana* [67]. Human TREX-2 consists of GANP (Sac3 in yeast), ENY2 (Sus1), CETN2/CETN3 (Cdc31), PCID2 (Thp1), and DSS1 (Sem1). ENY2 is also a component of SAGA [68]. Recent findings indicate that GANP can interact with Nxf1 and facilitate mRNA export associated with Nxf1 [69,70], and another study showed that GANP is required to export both intronless and spliced mRNA [71]. TREX-2 associates with the NPC via the nucleoporins TPR and Nup153. Moderate nuclear accumulation of poly(A) RNA occurs when any one of Nup153, TPR, GANP, or ENY2 is knocked down [68]. Therefore, human TREX-2 appears to facilitate the interaction between mRNA transcription sites and the NPC and to promote mRNA export. It is not clear whether SAGA and TREX-2 in higher eukaryotes function like the proteins in yeast, although several studies have demonstrated that some mobile Nups can interact with active transcription sites and regulate expression of their target [72,73,74].

### 2.4. Crm1-Dependent mRNA Export

Nxf1 functions in bulk mRNA transport, whereas rRNAs, U snRNAs, and a certain subset of mRNAs are exported in a Crm1-dependent manner [75,76]. The export of signal recognition particle (SRP) RNAs also depends on Crm1 in *S. cerevisiae* [77,78], whereas in vertebrates, SRPRNAs are exported by exportin-5, which is known as the export receptor of tRNA [79]. The role of Crm1 as an RNA export receptor was first revealed by the report that Crm1 exports unspliced mRNA of HIV in a mechanism dependent on Rev, a viral RNA binding protein [76,80,81]. Crm1 is a member of the importin-beta family, and is well known as a protein export receptor; however, it needs an adaptor protein to interact with RNA because it is not an RNA binding protein. Several Crm1 adaptor proteins have been identified.

**Figure 3:**
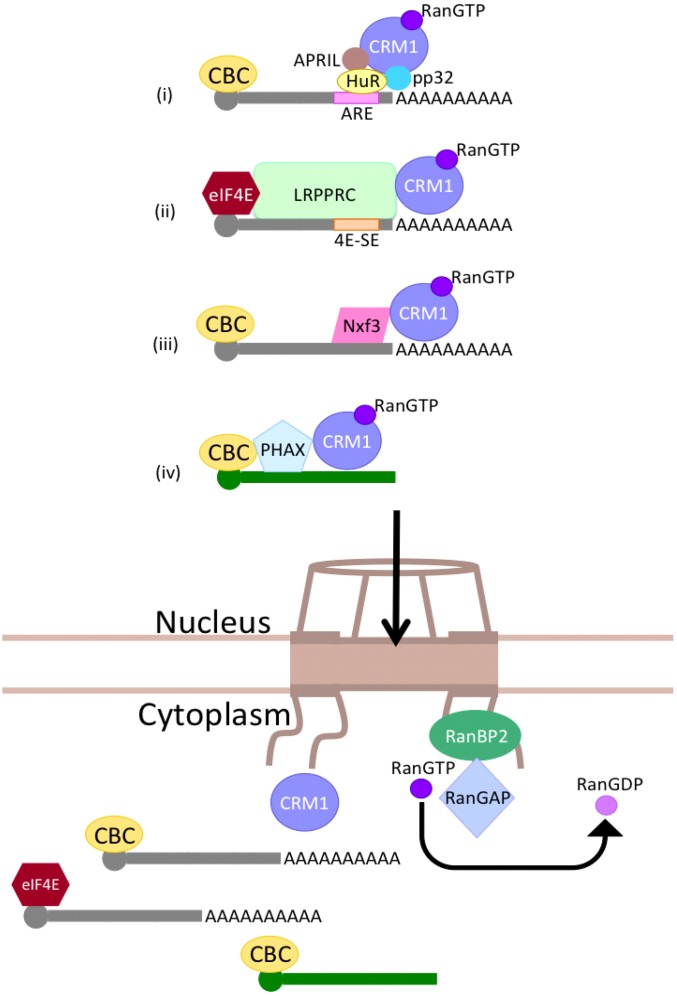
Crm1-dependent mRNA export. Several subsets of mRNA are exported by Crm1. There are four known pathways: (i) HuR-mediated; (ii) LRPPRC-mediated; (iii) Nxf3-mediated; and (iv) U snRNA export. Each pathway requires binding of Crm1 and RanGTP. Hydrolysis of GTP by RanGAP on the cytoplasmic face of the NPC releases mRNA into the cytoplasm.

To date, three adaptor proteins have been shown to function in the Crm1-dependent mRNA export pathway: RNA-binding protein human antigen R (HuR), leucine-rich pentatricopeptide repeat protein (LRPPRC), and nuclear export factor 3 (Nxf3) (Figure 3). HuR can bind RNA containing an AU-rich element (ARE). In this pathway, Crm1 binds pp32 and APRIL in addition to HuR to export this class of RNA [82]. LRPPRC interacts with Crm1, eIF4E, and the RNA element 4E-SE [83]. Nxf1 and AlyRef are not involved in this LRPPRC- and eIF4E-dependent mRNA export pathway. Nxf3 is a member of the Nxf family, but lacks the *C*-terminal domain that is required for binding of Nxf1 to the NPC. Instead, Nxf3 possesses a Crm1-dependent export signal and can tether RNA, and therefore functions in the Crm1-dependent RNA export pathway [84]. In mice, Nxf3 is expressed in Sertoli cells but seems to be dispensable [85]. There are also many RNAs exported by Crm1 for which the adaptor proteins are currently unknown [86].

Like mRNA, spliceosomal U snRNAs are transcribed by RNAPII and have the same m^7^G-cap and CBC [87]. However, instead of AlyRef, the adaptor protein PHAX binds to both the CBC and near the cap of U snRNA. PHAX subsequently recruits Crm1-RanGTP, leading to export of the U snRNA [88]. The high affinity of PHAX for small RNAs of less than 200–300 nt distinguishes this RNA export pathway [89].

### 2.5. Passage of mRNP with Crm1 through the NPC

Many factors engage in mRNP transport via Crm1, including GTP binding Ran (Ran-GTP), RanBP1, RanBP2 (Nup358), RanBP3, and RanGAP. Crm1 interacts with the nuclear export signal (NES) of its cargo in a RanGTP-dependent manner and RanBP3 supports this interaction [90]. Crm1 can interact with FG repeat-containing Nups and pass through the central channel of the NPC, thus Crm1-bound RNA can be transferred to the cytoplasmic face. RanBP2 is associated with the cytoplasmic face of NPC, whereas RanBP1 and RanGAP are soluble in the cytoplasm. GTP hydrolysis is required to dissociate RNA from export receptors. Interaction with RanBP1 and RanGAP promotes this hydrolysis and subsequent decoupling [91]. RanBP2 has binding capacity with Nup88, Nup214, RanGAP, RanGTP, RanGDP, and Nxf1-Nxt1. RanBP2 performs a similar function to RanBP1 in the dissociation of mRNP from RanGAP [91,92,93,94,95,96]. It is unknown whether DDX19 functions in the remodeling of the Crm1-dependent RNA export pathway.

Interestingly, RBM15 promotes the export of mRNAs containing the *cis*-acting RNA transport element [49], and this element is exchangeable for the viral Rev and Rev response element (RRE) system [97]. Therefore, RBM15 might participate in Crm1-dependent mRNA export.

## 3. rRNA

The ribosome that serves as the machinery of protein synthesis (translation) is a large complex that consists of two subunits formed by ribosomal RNAs (rRNAs) and ribosomal proteins (RPs). The small subunit (the 40S subunit) contains 18S rRNA, and the large subunit (the 60S subunit) contains 5S, 5.8S, and 28S (25S in yeast) rRNAs [98]. All rRNAs except for 5S are transcribed by RNA polymerase I (RNAPI); 5S RNA is transcribed by RNA polymerase III (RNAPIII) [99]. In mammals, all rRNAs transcribed by RNAPI are produced from the single nascent pre-rRNA by the combination of endo- and exo-nucleolytic processing. Subsequent to transcription, each premature rRNA interacts directly with RPs in the nucleus and forms a pre-60S or pre-40S subunit. These subunits are exported and processed in the cytoplasm.

In *S. cerevisiae*, the pre-60S subunit is exported by Crm1 [100,101], Mex67-Mtr2 [102], or Arx1 [103]. The Mex67-Mtr2 complex and Arx1 can bind the pre-60S subunit directly, whereas Crm1 needs the adaptor protein Nmd3 to interact [104]. The pre-40S subunit is exported by Crm1 [105] or Mex67-Mtr2 [106]. Ltv1 and Pno1/Dim2 are regarded as adaptors for the interaction with pre-40S subunit and Crm1 [107,108,109]. In addition, both subunits require the assembly factors Rrp12 and Sda1, which harbor HEAT-repeat motifs, for the nuclear export of pre-40S rRNA [105,110,111].

In mammals, the pre-60S subunit is exported by Crm1 [112] or Exportin 5 (Xpo5) [113], whereas the pre-40S subunit is exported by only Crm1 [112,114]. Nmd3 functions in the export of pre-60S with Crm1 [112]. It remains unclear whether Nxf1, a homolog of Mex67, functions as the nuclear export receptor for rRNA.

The export of pre-ribosomes is closely related to their proper assembly. Therefore, it is often difficult to distinguish between the factors that are required to complete processing of pre-ribosomes in the nucleus and those that facilitate the nuclear export of pre-ribosome.

## 4. tRNA

**Figure 4:**
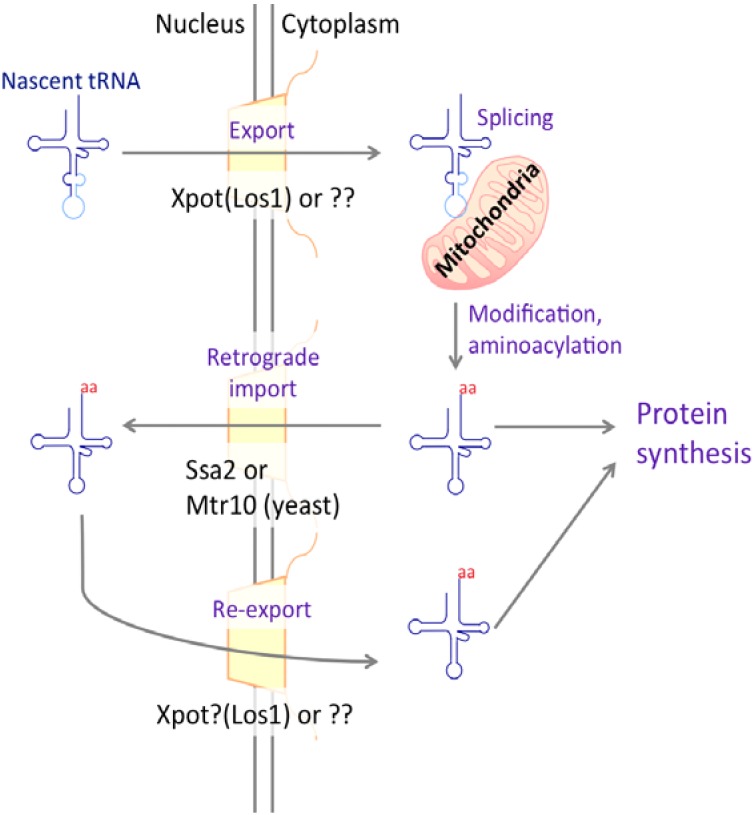
Transportation of tRNA. Nascent tRNA is exported to the cytoplasm and processed into mature tRNA, which remains in the cytoplasm and can function in protein synthesis. However, a tRNA retrograde import pathway operates under some stress conditions. There are several known tRNA export receptors in yeast (Los1, Ssa2, and Mtr10), and Xpot has been identified in humans.

Transfer RNA plays an essential role in translation by transporting amino acids to the ribosomal complex for peptide chain elongation. tRNA genes are scattered throughout the human and yeast genomes, and are transcribed by RNAPIII. At M phase of the cell cycle, the transcriptional level of tRNA peaks because of the association of tDNA with Nup2 and Nup60 via Cohesin [56]. Interestingly, artificial association of Nups and tDNA can increase the transcriptional level in other phases. tRNAs are exported to the cytoplasm for various modifications (e.g., aminoacylation). Interestingly, they can be trafficked from the cytoplasm to the nucleus via the tRNA retrograde process [115,116,117,118,119], and subsequently re-exported to the cytoplasm [120,121] (Figure 4). This shuttling is conserved in vertebrates [122,123,124].

### 4.1. Export Receptors of tRNA

A export receptor is also required for tRNA to cross the NPC meshwork [125]. The main transport factor for tRNAs is exportin-t (Xpot) in vertebrates and its ortholog Los1 in *S. cerevisiae* [126,127,128]. Xpot is a member of the importin-beta family and, like Crm1, is regulated by the small GTPase Ran.

The yeast los1∆ strain shows nuclear accumulation of end-processed intron-containing tRNAs. A defect in tRNA export results in such a phenotype because the splicing machinery for tRNA is located on the outer surface of the mitochondria [116,129,130]. At the present time, Los1 is the only protein known to transport intron-containing pre-tRNA into the cytoplasm in *S. cerevisiae* [131]. However, the yeast los1∆ strain is viable. Likewise, the *A. thaliana* Xpot homolog PAUSED is nonessential, and insects lack a Xpot homolog [132,133,134,135,136]. Taken together, these data suggest the existence of a novel nuclear exporter for tRNA. Incidentally, some studies have shown that Los1 contributes to tRNA modification in *S. pombe* [137,138,139,140].

The importin-beta family member Msn5/exportin-5 (Xpo5) is also postulated to be involved in tRNA export in yeast and *Drosophila* [119,131,141]. In contrast, vertebrate Xpo5 principally exports miRNA to the cytoplasm, thus the role of Xpo5 in tRNA export is assumed to be minor [142,143,144,145]. A *S. cerevisiae* msn5∆ strain shows nuclear accumulation of intronless tRNA, but not intron-containing tRNA [131]. Consequently, Msn5 probably functions in the export of intronless pre-tRNAs and the re-export of mature tRNA.

### 4.2. tRNA Retrograde Nuclear Import

After transcription and export, tRNA is modified and contributes to translation in the cytoplasm. However, tRNA can also undergo retrograde import into the nucleus. This process is conserved from yeast to humans [122,123,146]. Adaptors that function in this retrograde import in yeast are Ssa2 and Mtr10. Mtr10 requires Ran for tRNA import [118], whereas Ssa2 seems to transport tRNA with heat shock protein in a Ran-independent manner [119,147]. The biological function of this retrograde import is speculated to be tRNA modification or tRNA quality control [147,148]. In fact, aminoacylation of tRNA occurs not only in the cytoplasm, but also in the nucleus [115,116,117,147]. This retrograde pathway is accelerated when the cells are starved [118,120,147].

## 5. miRNA

MicroRNAs are small RNA molecules of approximately 22 nt that regulate gene expression via RNA silencing. miRNA is processed in both the nucleus and the cytoplasm; therefore, the miRNA traversing the NPC is a precursor miRNA (pre-miRNA) rather than a fully processed miRNA.

**Figure 5:**
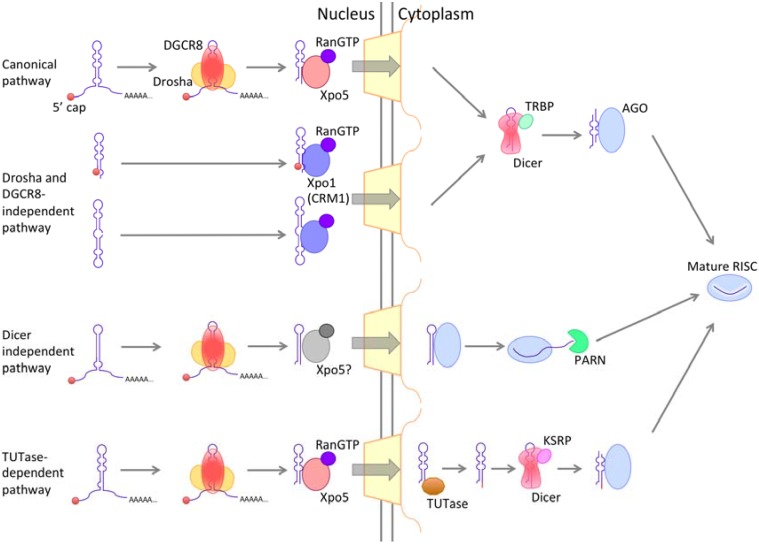
MicroRNA export pathways. Nascent miRNA is processed in both the nucleus and the cytoplasm. The nuclear export receptor involved in the Dicer-independent pathway is unknown.

Most of the miRNA is transcribed as primary miRNA (pri-miRNA) by RNAPII in the nucleus, and pri-miRNA has a 5' cap, poly(A) tail, and local hairpin structure [149,150]. Mature miRNA sequences are embedded in this stem-loop structure. The nuclear RNAse III Drosha and its essential cofactor DGCR8 crop unnecessary stem-loop and ssRNA regions. Completion of this processing allows the pre-miRNA to be exported [151,152,153,154,155]. Exportin 5 (Xpo5 or Exp5) functions in the export of pre-miRNA with RanGTP, similar to Crm1 and Xpot [144,156,157]. It is not known whether Xpo5 requires another cofactor. Xpo5 knockdown results in a decrease in miRNA level in the cytoplasm, but curiously does not result in miRNA accumulation in the nucleus [157]. Cell cycle progression and cell proliferation are intimately related to miRNA export. Xpo5 is expressed ubiquitously and can be induced during cell cycle entry through a PI3K-mediated mechanism [158]. In some tumors, Xpo5 is mutated to a C-terminal truncated form. This mutant Xpo5 cannot transport pre-miRNA cargo, resulting in a global reduction in the level of mature miRNAs [159]. After transfer into the cytoplasm, pre-miRNA is further processed to become mature miRNA (Figure 5). Pre-miRNA that is exported to the cytoplasm is processed by Dicer and loaded onto AGO protein to form the RNA-induced silencing complex (RISC). miRNA functions in RNA silencing through the formation of RISC.

The pathway described above is a canonical pathway; in addition, three non-canonical pathways have been described, a Drosha- and DGCR8-independent pathway; a Dicer-independent pathway; and a TUTase-dependent pathway [160]. For the Drosha and DGCR8-independent pathway, trimming of the ssRNA regions of pre-miRNAs in the nucleus is not necessary. It has been suggested that Crm1 is the nuclear exporter of this pathway, and that several pre-miRNAs without an m^7^G-cap are exported in this pathway (e.g., miRNA-like small RNA in ACA45 snoRNA) [161,162,163,164]. The Dicer-independent pathway is involved in the biogenesis pathway of miR-451 [165,166,167]; pre-miR-451 is not cut by Dicer, but it is directly loaded onto AGO2 protein and is cleaved by AGO2 [165], and then a poly(A)-specific ribonuclease, PARN, trims pre-miRNA-451 on AGO2 to produce mature RISC. The TUTase-dependent pathway is used for several group II pre-miRNAs (e.g., the let-7 family in vertebrates) [168]. These RNAs have a 3' overhang that is one nucleotide shorter than that of general pre-miRNAs and therefore needs to be extended by one nucleotide by terminal uridylyl transferases (TUT) to undergo Dicer-mediated processing.

## 6. Viral mRNA Export

Viruses have evolved special mechanisms of gene expression to replicate. Most steps of viral gene expression depend on the machineries of its host cell and mRNA export is no exception. When a viral genome is replicated, some species retain their genome in the cytoplasm of the host cell, whereas others transport their genome into the nucleus. In the last section of this review, we discuss viral mRNA export, focusing on two retroviruses, MPMV and HIV. We will describe viral replication with a focus on the nucleocytoplasmic transport of RNA in accordance with the concept of this review. A retrovirus usually has a single promoter in its whole genome, which means it has just one primary transcript [169]; however, multiple types of viral mRNA and genome RNA exist in the cytoplasm of the host cell. 

This is achieved by export of the viral mRNA in either intron-containing or spliced forms. Usually, intron-containing mRNA cannot translocate into the cytoplasm because of the mRNA quality control system. The retrovirus has special systems that enable the export of intron-containing mRNA, thus overcoming the quality control system.

### 6.1. mRNA Export Pathway of MPMV

MPMV has two different mRNA export pathways: a splicing-dependent pathway and a splicing-independent pathway. The former is a simple pathway in which MPMV mRNA is exported to the cytoplasm using the bulk mRNA export pathway of its host cell. The MPMV primary transcript is recognized by the spliceosome, which presumably results in the sequential recruitment of TREX-1 and then the transporter Nxf1. The spliced MPMV mRNA that is exported in this way encodes the Env protein. In contrast, the splicing-independent pathway translocates MPMV mRNA to the cytoplasm in its intron-containing form. This pathway depends on the constitutive transport element (CTE), an RNA element located in a 3' untranslated region of the MPMV primary transcript [170,171]. CTE has a stem loop structure that consists of a 9-nt terminal hairpin loop and two identical 16-nt internal loops [171,172]. The two 16-nt internal loops directly recruit Nxf1, resulting in mRNA export without splicing [173,174]. In CTE-dependent mRNA export, the RNP domain and LRR domain of Nxf1 are required for the recruitment to CTE. The RNP domain of Nxf1 is sufficient for the bulk mRNA export pathway [175]. Structural analysis suggests that the interaction among the CTE, RNP domain, and LRR domain of Nxf1 resembles the U2B"-U2A' spliceosomal heterodimer in structural and biochemical properties, and efficiently functions in the recruitment of Nxf1 to CTE RNA [176]. In this way, MPMV produces two forms of mRNA.

The interaction of Nxf1 with the CTE has also reported using endogenous *Nxf1* mRNA and Nxf1 proteins [177]. The nascent transcript of *Nxf1* contains the CTE in its alternatively spliced intron. This CTE-containing mRNA is translated into truncated Nxf1, the function of which is unknown.

### 6.2. mRNA Export Pathway of HIV

**Figure 6:**
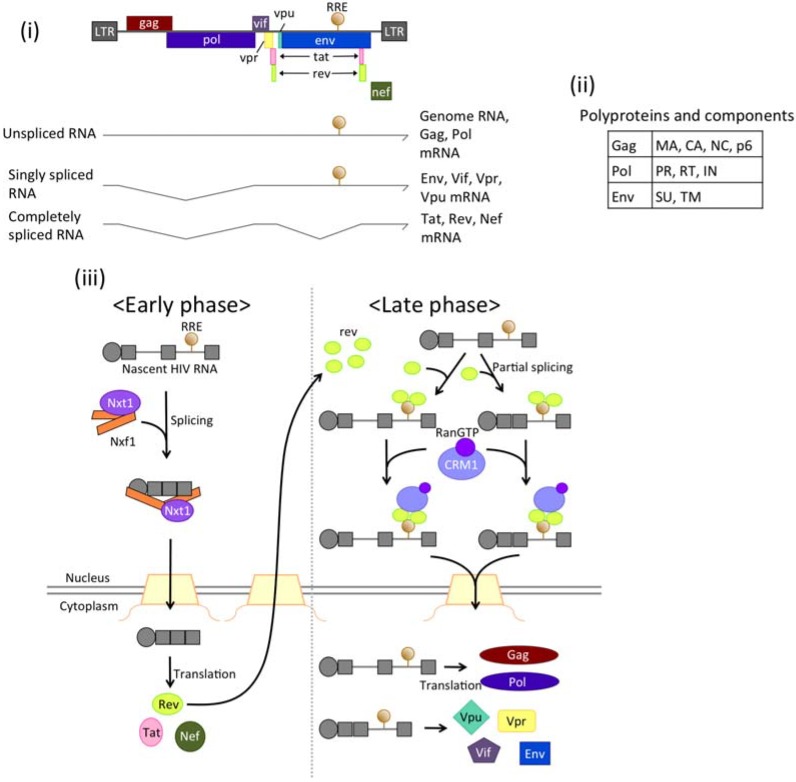
(**i**) Structure of the HIV-1 genome. The genome of HIV-1 encodes 15 proteins and a genomic RNA, which are all derived from a single transcript; (**ii**) The components of three polyproteins, Gag, Pol, and Env; (**iii**) mRNA export in HIV-1. In the early phase, transcripts of HIV-1 are exported into the cytoplasm in a splicing-dependent manner. mRNAs exported in this way encode proteins including Rev. Rev protein is localized in the nucleus, where it is bound to RRE, which is present in an intron. The nuclear export receptor Crm1 is recruited to Rev. As a result, intron-containing mRNAs are exported to the cytoplasm.

HIV-1 has evolved a more complicated and sophisticated mechanism of mRNA export than MPMV. HIV-1 produces 15 proteins, including regulatory and accessory proteins in addition to the structural proteins, enzymatic proteins, and envelope proteins [178]. HIV-1 has Rev and RRE-dependent and -independent mRNA export pathways, although HIV controls both pathways more strictly (Figure 6).

In the early phase, HIV-1 mRNAs are exported into the cytoplasm in a splicing-dependent manner. This type of mRNA is completely spliced and encodes two regulatory proteins, Tat and Rev, and one accessary protein, Nef [179]. The Rev protein provides the other mechanism of mRNA export, which occurs dependently on Rev and RRE in the late phase. Rev protein is reported to shuttle between the nucleus and the cytoplasm [180]. As soon as Rev protein is translated, it localizes in the nucleus where it directly binds to HIV-1 mRNA via the Rev response element (RRE), a 221-nt RNA element with six stem loop structures that is located in an intron of HIV mRNA [181,182,183,184]. Rev proteins multimerize on RRE, and then Crm1 interacts with a leucine-rich NES in Rev. Crm1 forms a dimer upon Rev-RRE binding and is essential for the export of HIV-1 mRNA [185]. Unspliced HIV-1 mRNA is exported by Crm1, eventually packaged as an HIV-1 genome RNA by envelope proteins. Mutational analyses have revealed that this multimerization of Rev is essential for the recognition of RRE [181], whereas only the 34-nt stem loop II B structure is necessary and sufficient for its recognition by Rev *in vitro* [184,186]. Structural analyses have revealed that the entire structure of the RRE is required for optimal RRE function [187]. According to these analyses, RRE adopts an “A”-like structure in which the legs constitute two binding sites for Rev and are positioned ~55 Å apart [183], matching the width of the Rev dimer. When Rev protein is bound to the RRE it interacts with CBC, thus suppressing the recruitment of TREX-1 and Nxf1-dependent export of RRE-containing mRNA [188].

By taking advantage of the mRNA export machinery of its host cell, HIV-1 exports as many as seven isoforms of mRNA derived from one primary transcript, thus producing multiple proteins.

## 7. Conclusions

This review summarizes a broad range of RNA export mechanisms through the NPC. In general, RNA export receptors are selected according to the type of RNA, but there are many overlapping relationships between the type of RNA and the export receptor used. Studies using yeast are much more advanced than those using human or other mammalian cells, especially with respect to the interaction between RNA-protein complexes and the NPC components. Therefore, further studies of the RNA export mechanisms in humans are required in the future. There are two interesting studies reporting export of RNA-containing particles in an NPC-independent manner. In both cases, RNA export is achieved by nuclear envelope budding. One involves the egress of herpesvirus nucleocapsid [189] and the other is the export of large RNP granules harboring synaptic protein transcripts at the *D. melanogaster* larval neuromuscular junction [190]. We presume that such alternative RNA export pathways will attract more attention in the future.

Studies into the mechanism of mRNA export contribute to progress in various fields of research, such as viral infectious diseases, cancer, and efficient protein production systems using mammalian cells. Moreover, a lot of attention has been paid to the relationship between the defects in RNA metabolism and neurodegenerative diseases [191]. Insights into RNA transport and metabolism are potentially applicable to medical care and industrial applications.
